# Changes in microbiota during experimental human Rhinovirus infection

**DOI:** 10.1186/s12879-015-1081-y

**Published:** 2015-08-14

**Authors:** J. J. Hofstra, S. Matamoros, M. A. van de Pol, B. de Wever, M. W. Tanck, H. Wendt-Knol, M. Deijs, L. van der Hoek, K. C. Wolthers, R. Molenkamp, C. E. Visser, P. J. Sterk, R. Lutter, M. D. de Jong

**Affiliations:** Department of Medical Microbiology, Academic Medical Centre, University of Amsterdam, Meibergdreef 9, 1105 AZ Amsterdam, The Netherlands; Department of Experimental Immunology, Academic Medical Centre, University of Amsterdam, Amsterdam, The Netherlands; Department of Respiratory Medicine, Academic Medical Centre, University of Amsterdam, Amsterdam, The Netherlands; Department of Clinical Epidemiology, Biostatistics and Bioinformatics, Academic Medical Centre, University of Amsterdam, Amsterdam, The Netherlands; Department of Anaesthesiology, Academic Medical Centre, University of Amsterdam, Amsterdam, The Netherlands

## Abstract

**Background:**

Human Rhinovirus (HRV) is responsible for the majority of common colds and is frequently accompanied by secondary bacterial infections through poorly understood mechanisms. We investigated the effects of experimental human HRV serotype 16 infection on the upper respiratory tract microbiota.

**Methods:**

Six healthy volunteers were infected with HRV16. We performed 16S ribosomal RNA-targeted pyrosequencing on throat swabs taken prior, during and after infection. We compared overall community diversity, phylogenetic structure of the ecosystem and relative abundances of the different bacteria between time points.

**Results:**

During acute infection strong trends towards increases in the relative abundances of *Haemophilus parainfluenzae* and *Neisseria subflava* were observed, as well as a weaker trend towards increases of *Staphylococcus aureus*. No major differences were observed between day-1 and day 60, whereas differences between subjects were very high.

**Conclusions:**

HRV16 infection is associated with the increase of three genera known to be associated with secondary infections following HRV infections. The observed changes of upper respiratory tract microbiota could help explain why HRV infection predisposes to bacterial otitis media, sinusitis and pneumonia.

**Electronic supplementary material:**

The online version of this article (doi:10.1186/s12879-015-1081-y) contains supplementary material, which is available to authorized users.

## Background

Human Rhinovirus is a globally endemic virus, belonging to the family of Picornaviridae and the most common cause of the common cold. Although usually causing mild to moderate disease, HRV triggers exacerbations of chronic obstructive pulmonary disease, asthma and cystic fibrosis [1, 2], and may cause severe lower respiratory tract disease particularly in risk groups such as infants, the elderly and immunocompromised patients [3]. In addition, infection with HRV is a predisposing factor for secondary bacterial infections (otitis media, sinusitis, pneumonia) [4–7]. Insights into the mechanisms through which HRV enhances bacterial supra-infection remain limited.

HRV associated bacterial superinfections are assumed to be caused in part by anatomical and mechanical causes, e.g. due to Eustachian tube dysfunction [8], ostiomeatal obstruction and reduced mucocilliary clearance [9, 10]. In addition, HRV infection may increase epithelial permeability [11], promote bacterial adherence and entry into the epithelium [12, 13] or reduce host responses to bacterial pathogens[14]. Common pathogens associated with HRV-associated bacterial infections include *S. pneumoniae*, *H. influenzae* and *M. catarrhalis*.

In recent years, culture-independent DNA-based techniques demonstrated that highly complex microbial communities are present in the upper airways. These communities are an integral part of our commensal airway microbiota and likely contribute to infection and disease. HRV infection and the subsequent host response [11–14] change the upper airway local environment and will likely alter the microbiota composition. Insights into HRV induced changes in microbiota composition could contribute to our understanding of the pathophysiological mechanisms of bacterial super-infection and could help to predict secondary bacterial infections thereby ultimately contributing to preventive strategies and tools. Most human studies to date have been retrospective, showing epidemiological correlations between HRV and the presence of potential respiratory pathogens [15–17]. Such correlation, however, do not necessarily implicate causality and interpretation of these data warrants caution. Most studies of the lung microbiota have been cross-sectional, observational and descriptive in nature [18].

Here we describe the effects of HRV infection on the upper respiratory tract microbiota in otherwise healthy human volunteers experimentally infected with HRV serotype 16 (HRV16), enabling controlled observations before, during and after resolution of infection. We hypothesized that HRV16 infection alters throat bacterial microbiota compositions in healthy volunteers in terms of the bacterial diversity (Shannon and Simpson diversity indexes) as well as bacterial richness (Chao1 measure). We also analysed changes over time in relative abundances of individual bacterial genera/species.

## Methods

### Study subjects

Healthy volunteers (6 in total) aged 18–60 were eligible for participation provided they had not had a common cold during the six weeks prior to enrolment.

Presence of common cold was defined as a cumulative validated cold symptom score [19] of ≥ 14 over a 6 day period and the subjective impression of a cold, rhinorrhoea on at least three days. Other exclusion criteria were: receipt of BCG-vaccination, chest x-ray abnormalities, a history of lung disease, signs of airway obstruction (Forced Expiratory volume in 1 s (FEV1) > 80 % of predicted value), seasonal or perennial rhinitis or sinusitis, smoking (unless stopped smoking 12 months prior to the study, max. 5 pack years), any clinically significant abnormality in medical history and clinical examination, participation in any clinical investigational drug treatment protocol within 30 days of enrolment, pregnancy or lactation, any medication usage. The study protocol was approved by the Academic Medical Center Amsterdam ethical review committee. Written informed consent was obtained from all volunteers.

### Study design

**Fig. 1:**
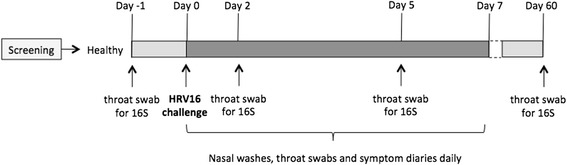
Study design. Screening was performed on the day before experimental rhinovirus infections. Nasal washes and throat swabs for Human Rhinovirus (HRV) PCR and viral culturing were repeated daily from day 0 until day 7. A throat swab for 16S rRNA gene sequencing was collected prior to HRV16 challenge (day -1) and subsequently on days 2, 5, and day 60

In this prospective single center study all volunteers were experimentally infected with HRV16 (Fig. 1). Upon enrolment the volunteers were screened to ensure the absence of circulating antibodies against tuberculosis, HRV16, Human T-lymphotropic virus Type 1 (HTLV-1) and 2 (HTLV-2), Human Immunodeficiency virus (HIV), and Hepatitis A, B and C virus (HAV, HBV, HCV). Acute infection with Influenza A and B, enterovirus sp., adenovirus sp., rhinovirus sp., human metapneumovirus, human respiratory syncytial virus sp., parainfluenza viruses 1-4, human parechovirus, human bocavirus, coronavirus sp., *Chlamydia pneumoniae*, *Mycoplasma pneumoniae*, *legionella sp.* was excluded through validated PCR assays on nasal lavage and throat swabs. These throat swabs were collected to serve as baseline microbiota measures. One day later volunteers were experimentally infected with HRV16 (details below), which has been shown to cause mild common-cold symptoms. Nasal lavages and throat swabs were performed daily for 7 days. On day 60 a convalescent throat swab was taken. All volunteers were requested to report common cold and asthma symptoms daily until day 7 after rhinovirus challenge using a standardized cold symptom score [20]. Diaries were collected at the final visit.

### HRV16 challenge

Experimental HRV16 infection was induced as described previously [21]. In short, 100 μl of an HRV16 aliquot (stored at -80 °C) was kept on ice, thawed and diluted in sterile 0.9 % NaCl just before exposure. 750 μl of this dilution was transferred to a sterile micronic tube positioned in a glass support of the DeVilbiss 286 atomizer (DeVilbiss Healthcare, Somerset, PA, USA). This dilution was administered to the healthy volunteer by spray in one nostril while closing off the other nostril. The total administered HRV16 dose was 10TCID_50_. Infection was confirmed by positive PCRs, positive viral cultures and development of symptoms.

### Nasal lavage

One nostril was closed off using an inflatable catheter as described previously [20].

Slowly, 10 ml of sterile Hanks balanced salt solution (without phenol) at body temperature was instilled via the catheter and left for 5 min. Lavage fluid was collected in tube for viral detection. The procedure was repeated in the other nostril. All used materials were sterile or disinfected using 70 % ethanol prior to use.

### Throat swabs

A cotton-tipped swab (Copan Diagnostics, Corona, Canada) was used to obtain material from the space between the palatine arches. The cotton swab was transferred to universal transport medium (UTM, Copan Diagnostics) in a tightly closed tube. After vortexing, the swab was discarded and the medium was stored at -80 °C.

Infection was confirmed by HRV-positive quantitative PCR in nasal and/or throat samples as described elsewhere [19]. In addition, presence of replication-competent HRV was confirmed by virus culture (human embryonic lung cells).

### 16S rRNA gene analysis

After treatment with an in-house enzymatic cocktail (achromopeptidase, mutanolysine, lysostaphine (1000:100:3) and lysosyme (1 mg/ml) (Sigma-Aldrich, St. Louis, MO, USA) in TE-buffer) as well as an in-house lysis buffer (Sodium-docecyl-Sulphate (1 %), Tween-20 (0.5 %) and Sarkosyl (0.5 %) in TE-buffer), DNA was extracted using the MagnaPure LC (Roche Diagnostics, Indianapolis, IN, USA) following the manufacturer’s instructions. Bacterial rRNA genes were quantified by rt-PCR using primers F-16S-27 (5′-AGAGTTTGATCCTGGCTCA G-3′) and R-16S-355 (5′- GCTGCCTCCCGTAGGAGT-3′) which bind to an area of the 16S rRNA gene conserved in all bacteria [26, 27], and probe P-16S (6FAM-CTGGCGGCRKGCYTAACACATGCAAGTCGA-BHQ1). Subsequently, 16S rRNA genes were amplified using the primers detailed in the supplementary material followed by a second round of PCR using primers with adapters and barcodes (see Additional file 1 for full description).

The amplified fragments with adapters and tags were quantified using the Quant-iT dsDNA Assay Kit on a Qubit fluorometer (Invitrogen/Life Technologies, Carlsbad, CA, USA). Emulsion PCR was performed according to the protocol (emPCR Method Manual – Lib-A SV jan-2010) supplied with the GS FLX Titanium XLR 70 Sequencing kit (Roche Diagnostics, Indianapolis, IN, USA). Two- region 454 sequencing run was performed on a GS FLX Titanium Pico TiterPlate (70 × 75) using a GS FLX Titanium Sequencing Kit XLR70 according to the manufacturer’s instructions (Roche Diagnostics, Indianapolis, IN, USA).

### Data processing and analysis

Data analysis was performed using the software “Quantitative Insights into Microbial Ecology” (QIIME 1.8.0) [22]. After removal of low quality reads (quality score <25) and chimera (ChimeraSlayer) our data was denoised using the QIIME denoiser program [23]. Then we clustered the sequences into Operational Taxonomical Units (OTU’s) based on 97 % sequence similarity (Uclust) [24]. The resulting OTU table was then condensed by removing all OTUs representing less than 0.005 % of the total number of sequences [25]. Uclust classifier was used to assign taxonomy. FastTree was used to construct a phylogenetic tree for downstream analyses.

### Community structure

Bacterial taxonomic richness and diversity were determined using rarefaction plots of the normalized number of sequences per time point. Rarefaction curves were generated for 3 % genetic difference level (e.g. at the genus level). The Shannon diversity measure is a quantitative measure that reflects the number of different species simultaneously taking into account how evenly the bacteria are distributed among those species [26]. Another method to examine the microbiota community structure is to use the Chao1 measure which estimates the minimum richness in each sample [27]. Chao1, by taking into account the number of species found only once (singletons) or twice (doubletons) in a sample, provides an estimation of the number of undetected species in the microbiota, which is important as the human associated microbiota contains many rare species.

UniFrac is a method to calculate a distance measure between samples using the phylogenetic trees. The UniFrac measure expresses the fraction of shared branches in the phylogenetic trees between samples [28].

Ecological intra- and inter-patient microbiota similarity was estimated using adonis, ANOSIM and MRPP methods included in QIIME 1.8.0. Briefly, these methods compare the ecological distance between samples (Unifrac distance) to test the null hypothesis that intra-group similarity is not significantly different than inter-group similarity [29].

### Statistical analysis

With a sample size (n = 6) we expected to be able to detect an effect of 25 % on the bacterial richness measure Chao1 with two-sided significance of p = 0.05 and a power of 90 %, assuming a standard deviation of 20 %. We normalized the data by calculating the OTU proportion—the proportion of total sequences per microbial community, i.e., per sample. To compare differences in microbiota members between time points we used a linear mixed model. Microbial community comparisons were performed using parametric statistics in R, with the P values corrected by multiple hypothesis testing using the false discovery rate (FDR) [30]. To assess differences in overall community structure the data of all volunteers were pooled per time point and diversity measures (alpha diversity measures Shannon index, Simpson index and Chao1) [28]. Considering that the alpha diversity indices (i.e. Shannon diversity and Chao1) are sensitive to the original number of sequences generated from a given sample [31] we calculated the Shannon and Simpson diversity and Chao1 for normalized numbers of sequences for each separate sample. A number of randomly picked reads, corresponding to the lowest number of sequences in a sample group was picked 100 times from each sequence set.

## Results

### Clinical data

**Fig. 2:**
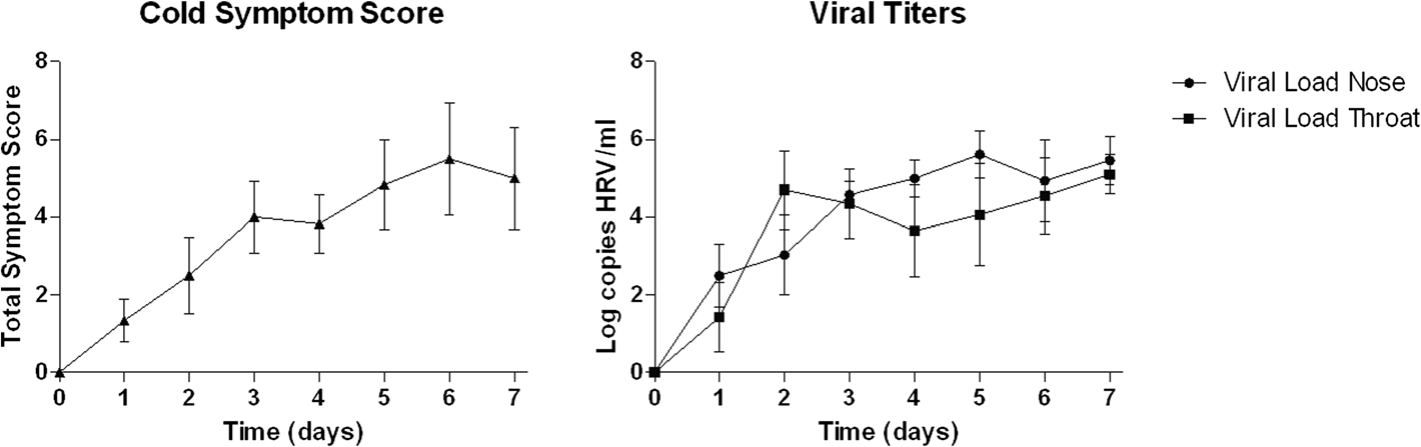
Human Rhinovirus Infection. Representation of the total Cold Symptom Scores as assessed by a daily questionnaire [1] as well as viral loads measured through RT-PCR in nasal lavage fluid and throat swabs of healthy volunteers after nasal inoculation of Human Rhinovirus serotype 16 (HRV-16)

In total 6 volunteers (3 males, 3 females) were included in the study (age range 18-28 years). HRV16 infection was established as demonstrated by positive HRV PCRs, positive viral cultures (data not shown) and development of symptoms in all. Viral RNA loads of nasal washes and throat swabs and the cold symptom scores are shown in Fig. 2.

### 16S analysis results

**Fig. 3:**
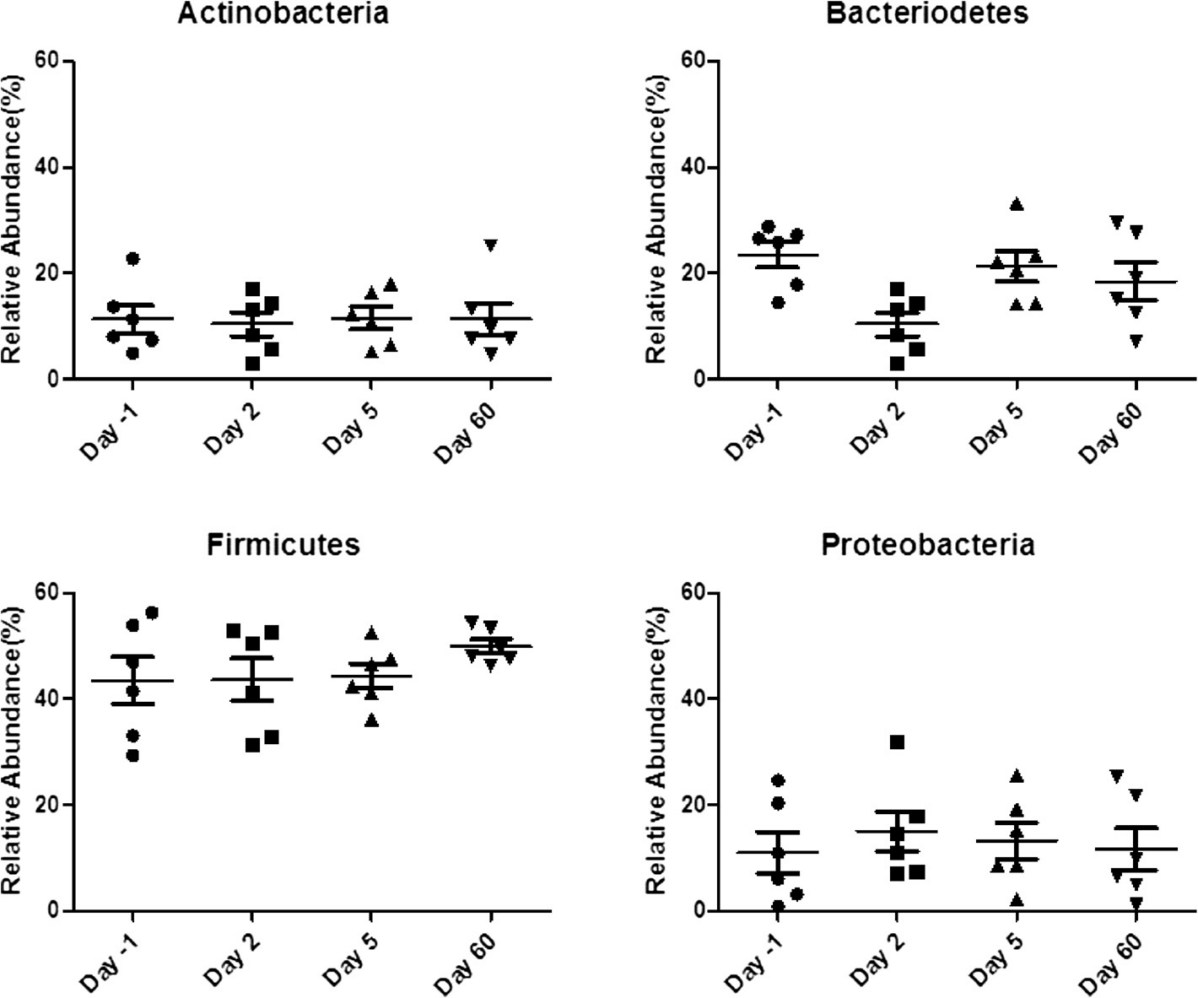
Bacterial Phyla. The distribution (and means ± SEM) of the top four phyla grouped by each time point: (day -1 (circles), day 2 (squares), day 5 (triangles pointing up), day 60 (triangles pointing down). No significant changes from baseline were observed in any of the phyla following HRV-16 infection (p > 0.05 across time points for all phyla using repeated measures ANOVA)

After removing sequences representing less than 0.005 % of the total abundance, the median number of sequences per sample was 5407 (min.: 2760; max.: 8589; sd: 1283.3). When sequences were clustered at 97 % sequence identity 384 operational taxonomical units (OTUs) were identified. Taxonomic analysis identified 11 bacterial phyla, containing 80 genera, 53 of which were successfully identified with >80 % confidence. The most commonly amplified bacterial phyla (Proteobacteria, Bacteroidetes, Actinobacteria, and Firmicutes) did not vary significantly over time (Fig. 3). *Streptococcus, Prevotella, Veillonella, Rothia*, and *Haemophilus* were the 5 most common bacterial genera amplified, together accounting for 63.8 % of the total number of sequences. These genera contained 101 species level OTUs, 20 of which were successfully identified (>90 % sequence similarity).

### Community structure

Alpha diversity measures (Chao1, Shannon and Simpson indexes) were similar in specimens obtained at baseline or convalescence (days -1 and 60) and during infection (days 2 and 5).

**Fig. 4:**
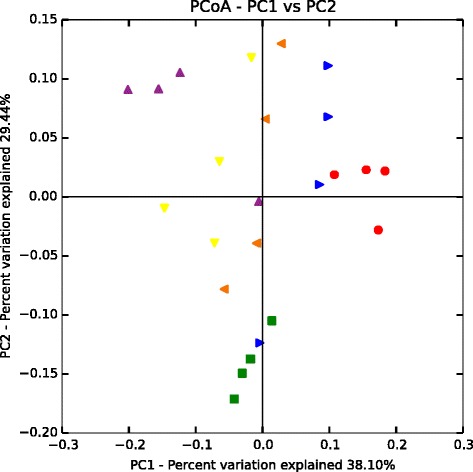
Principle component analysis (Unifrac measure). Samples coloured by individual: volunteer 1 (red triangles), volunteer 2 (blue triangles), volunteer 3 (brown triangles), volunteer 4(green circles), volunteer 5 (purple squares), volunteer 6 (yellow triangles). Generally samples seem to cluster by individual although there is substantial overlap between individuals. There were no significant changes over time with (weighted Unifrac significance). There were no significant differences between infected vs non-infected samples (weighted Unifrac significance)

A principle coordinates analysis based on the weighted UniFrac measure (fraction of shared branches in a phylogenetic tree) revealed that samples seem to cluster by individual volunteer although there was substantial overlap between individuals (Fig. 4). All 3 measures of ecological clustering (Adonis, ANOSIM and MRPP) showed that intra-individual UniFrac distance between samples was lower than inter-individual sample distance. Analysis on the data pooled by time point based on the UniFrac measure revealed no significant differences between time points (weighted UniFrac significance p > 0.05, *data not shown*) nor were time points 2 and 5 (during infection) significantly different from time points -1 (pre-infection) and 60 (convalescence). Using the UniFrac distance measure to assess changes in microbiota composition of each individual from its own baseline also revealed no significant differences between any time points (Fig. 4)

.

### Individual genera/species

Among the 5 most abundantly identified genera, three (*Streptococcus*, *Prevotella* and *Rothia*) showed very limited variation during HRV16 infection (less than 10 % of the baseline abundance), whereas *Veillonella* decreased by approximately 25 % and *Haemophilus* increased by 75 %.

**Fig. 5:**
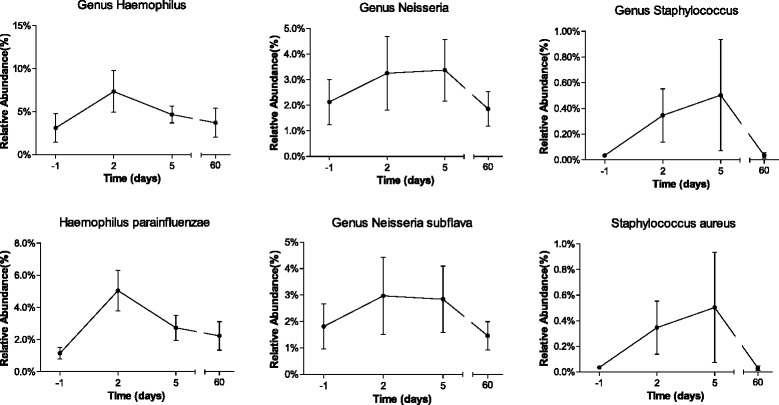
Changes over Time. Changes in relative abundances over time of the genera Haemophilus, Neisseria, Staphylococus and the bacterial species *H. parainfluenzae*, *Neisseria subflava* and *Staphylococcus aureus*. Strong trends towards increases in the relative abundance of the bacterial genera *Haemophilus* (raw p-value = 0.0041, false discovery rate (FDR) 0.178) and *Neisseria* (raw p-value = 0.0049, FDR 0.178) were observed, as well as a weaker trend towards increases of the bacterial genus *Staphylococcus* (raw p = value 0.0749, FRD 0.518). Analysis on species level revealed trends toward increases of *Haemophilus parainfluenzae* (raw p-value = 0.0098 FDR = 0.12), *Neisseria subflava* (raw p-value = 0.012 FDR = 0.12), *Staphylococcus aureus* (raw p-value = 0.0856 FDR = 0.245) during infection. Plots represent mean ± SEM

Paired comparisons using a linear mixed model were used to assess changes over time within individuals, days -1 and 60 being grouped as baseline non-infection measures, and days 2 and 5 being grouped as infection measures. No significant changes were observed between day -1 and day 60 specimens at genus or species level. However, at the genus level, a significant increase (p < 0.05) in relative abundance of *Haemophilus* and *Neisseria* was observed during infection. Analysis of these genera to species level show significantly increased *Haemophilus parainfluenzae* (p-value = 0.0098) and *Neisseria subflava* (p-value = 0.012). However, false discovery rate (FDR) correction for multiple comparisons showed an above threshold risk of type-I error for all these taxa (FDR = 0.12). Additionally, the largest fold-change observed was a 1200 % (13-fold) increase in the abundance of the genus *Staphylococcus (*p = 0.0749 FDR 0.518*)*, which only represents 0.2 % of the total sequences identified (Fig. 5). Analysis to species level shows a weak trend towards increases in *Staphylococcus aureus* abundance (p-value = 0.0856 FDR = 0.245).

The increases in *H. parainfluenzae* during infection were observed in all volunteers, while the *Neisseria* species increased in 5 of 6 volunteers. *Staphylococcus aureus* was only present in four volunteers and increased in three, hence the observed correlation between HRV16 and *Staphylococcus aureus* was the weakest (p-value = 0.0856 FDR = 0.245). Observed changes in the microbiota composition did not correlate with viral loads or symptom scores (data not shown).

## Discussion

In this study we demonstrate for the first time that infection with HRV16 in otherwise healthy volunteers alters upper respiratory tract microbiota composition. Although the overall changes are moderate, increases in relative abundances of the bacterial genera *Haemophilus, Staphylococcus* and *Neisseria* were observed. At a species level, increases were observed of *Haemophilus parainfluenzae*, *Staphylococcus aureus* and *Neisseria subflava* species. These increases in genera and species during HRV16 infection were observed in nearly all volunteers who carried these at baseline and relative abundances returned to baseline levels after the infection was cleared. Due to the limited number of study subjects (n = 6) and the stringent correction for multiple observations, these changes did not reach statistical significance. Nevertheless, it is striking that from over one hundred genera detected, substantial increases are recorded for 2 genera generally associated with secondary bacterial infections after HRV16 infection (*Heamophilus* and *Staphylococcus*).

These findings suggest that changes in the composition of resident microbiota per se may play a role in the development of bacterial super-infections associated with HRV infection. Our data extend previous findings by Molyneaux *et al.*, who described similar effects of experimental rhinovirus infection on the sputum microbiota in COPD patients [32] where in particular increases in *Haemophilus* sp. and *Neisseria* sp. were observed. However, in contrast with our findings, the microbiota of HRV infected healthy volunteers (i.e. without COPD) from the study by Molyneaux *et al.* did not seem to be affected [32] which may be explained by differences in study design, e.g. the use of sputum specimens vs. throat swabs. Interestingly, in asymptomatic Aboriginal children the presence of HRV was significantly associated with the presence of *H. influenzae* and *M. catarrhalis* in nasopharyngeal aspirates [15]. In a Dutch cohort of otitis-prone children, HRV positivity was significantly associated with the presence of *Streptococcus pneumoniae* and *M. catarrhalis* in the nasopharynx, but not with that of *H. influenzae* [17]. A later study in healthy children showed that *H. influenzae* was positively associated with the presence of HRV [16].

In vitro studies have demonstrated that infection with HRV induces changes in our microbiota’s habitat through augmentation of the adhesion of bacterial pathogens to the airway epithelium [11–13, 33, 34]. Given the complexity of the upper airway ecosystem it seems likely that direct viro-bacterial as well as bacterial-bacterial interactions may also play a role. Although a number of these direct interactions have been identified, their role during HRV infection remains to be elucidated [35]. The present new data on culture-independent classification of bacteria present in the throat during upper respiratory tract infection contributes to the understanding of upper respiratory tract microbial communities during HRV16 infection, and possible polymicrobial interactions.

Despite the statistical limitations mentioned above the observed trends occur in nearly all individual and involve ‘usual suspects’ for secondary bacterial infections. As the available volunteers for our study were relatively young extrapolation of these findings to other age groups should be done with caution.

Strengths of our study are the stringent exclusion of the presence of any other respiratory pathogens and our culture independent next generation sequencing approach, allowing sufficient depth to identify even the rarer species in our samples. However, deeper sequencing might have revealed minor other differences in the community composition [31]. Furthermore, our study was underpowered to investigate differences in individual bacterial species in the upper respiratory tract microbiota. Some limitations are inherent to 454 sequencing of 16S genes in general. They include possible bias introduced by the extraction, amplification and sequencing methodologies. Finally the number of genomic 16S copies varies considerably from bacteria to bacteria. Currently there is insufficient knowledge about exact 16S copy numbers to correct for this [36].

Although we were able to observe changes within each volunteer before, during and after HRV16 infection, we did not include a group of uninfected volunteers. However since our volunteers functioned as their own controls and the human microbiota during health has been shown to be relatively stable over time [37] it is likely that the observed microbiota changes were induced by HRV16 infection although we cannot exclude the possibility the daily collection of throat swabs itself may have affected microbiota composition. Our approach reduces the background noise inherent to human microbiota studies, which is mainly due to the large differences between individuals. This way we expect a more precise detection of relevant changes in the microbiota.

The fact that we used sequential samples allowed us to use the linear mixed model on non-independent (paired) samples, which focusses on the response of the variables of interest (bacterial taxa) during the course of the experiment and minimizes background noise of the high variability between individuals.

Our findings have clinical relevance and may contribute to further understanding of the early stages in the development of bacterial complications, such as acute otitis media, sinusitis and pneumonia, following HRV and other respiratory viral infections [4]. Larger controlled serially sampled studies in experimental and naturally occurring HRV infections are warranted to confirm and extend our current observations. It would be of clinical interest to investigate whether clinical secondary bacterial infections after HRV infection are actually preceded by similar changes in microbiota composition.

## Conclusions

In conclusion our study showed HRV16 infection is associated with strong trends towards increases in the relative abundances of *Haemophilus* and *Neisseria* and a weaker trend towards increases in the relative abundance of *Staphylococcus*. Even if the observed changes of upper respiratory tract microbiota are minor overall, they may be of great clinical significance and could help explain why HRV infection predisposes to bacterial otitis media, sinusitis and pneumonia.

## Additional file

Additional file 1:
**Bacterial 16S rRNA Gene Sequencing.** (DOCX 32 kb)

## Abbreviations

FDR: False Discovery Rate
FEV1: Forced Expiratory Volume in 1 s
HAV: Hepatitis A Virus
HBV: Hepatitis B Virus
HCV: Hepatitis C Virus
HIV: Human Immunodeficiency Virus
HTLV-1: Human T-lymphotropic virus Type 1
HTLV-2: Human T-lymphotropic virus Type 2
HRV: Human Rhinovirus
HRV16: Human Rhinovirus serotype 16
OTU: Operational Taxonomical Unit
QIIME: Quantitative Insights into Microbial Ecology
UTM: Universal Transport Medium
