# Supplementary material for: Changes in microbiota during experimental human Rhinovirus infection
Source: BMC Infect Dis. 2015 Aug 14;15:336. doi: 10.1186/s12879-015-1081-y (PMC4659412; doi:10.1186/s12879-015-1081-y)
Supplement: Additional file 1: — Bacterial 16S rRNA Gene Sequencing. (DOCX 32 kb) [file 12879_2015_1081_MOESM1_ESM.docx]

**Bacterial 16S rRNA Gene Sequencing**

*DNA extraction*

The throat swab samples we centrifuged for 15 minutes at 14000 rmp (Hettich Mikro 20 Centrifuge, Rotor Radius: 8.5). After disposal of the supernatant, the pellet was resuspended in 63 µl of Tris-EDTA (TE) medium. After adding 23 µl of enzymatic cocktail (achromopeptidase, mutanolysine, lysostaphine (1000:100:3) and lysosyme (1mg/ml) (Sigma-Aldrich, St. Louis, MO, USA) in TE-buffer)) the mixture was incubated at 37°C for 30 minutes on a shaking plate (800rmp). After adding 1 µl of proteinase K (50 U/ml, Roche diagnostics, Branford, CT, USA), 10 µl of an in-house lysis buffer (Sodium-docecyl-Sulphate, Tween-20 and Sarkosyl) was added following of incubation at 56°C for 120 minutes on a shaking plate (800 rpm). DNA extraction was performed using the MagnaPure LC (Roche Diagnostics, Branford, CT, USA).

*Quantification of bacteria*

Ribosomal DNA was quantified using real time PCR. Primers F-16S-27 (5’-AGAGTTTGATCCTGGCTCA G-3’) and R-16S-355 (5’- GCTGCCTCCCGTAGGAGT-3’) with probe P-16S (6FAM-CTGGCGGCRKGCYTAACACATGCAAGTCGA-BHQ1) which bind to an area of the 16S rRNA gene conserved in all bacteria [1, 2], were used along a probe (P-16S-univ-2) in a LightCycler 480 PCR System (Roche Diagnostics, Branford, CT, USA). After 2 min at 50°C the initial denaturation step was 95°C for 10 min, followed by 40 cycles of denaturation at 95°C for 15 s, annealing at 60°C for 60 s, and extension at 72°C for 60 s.

*16S rRNA gene amplification*

Samples were diluted to equal concentrations of 100pg/µl and amplified using the same primers used in the Q-PCR. 200 pg of extracted DNA was amplified by PCR. The reactions were performed using 20 µl (final volume) mixtures containing 10 µl of 10x LightCycler FastStart DNA Master SYBR Green I (Roche Diagnostics, Branford, CT, USA), 2 mM MgCl2, 0.9 µM of each primer, and 0.01U of Uracil N-glycosylase (Fermentas, Thermo Scientific, Waltham, MA, USA). The initial denaturation step was 95°C for 10 min, followed by 30 cycles of denaturation at 95°C for 15 s, annealing at 50°C for 60 s, and extension at 72°C for 60 s, followed by a final extension step that consisted of 72°C for 10 min.

To perform pyrosequencing, the product of the first amplification PCR was diluted in TE for amplification, and for adding barcodes and 454 adapters: in short a set of 24 primer sets (tags) was generated that each contained either the 27F or 355R 16S primers that were tagged with the 454 adapters. The forward primers were tagged at the 5 prime end with a 10 base ‘‘barcode.’’

PCR was performed on individual throat swab samples using the unique barcoded primers as follows: extracted 4 µl of the 100x diluted PCR product from the first amplification PCR was amplified by PCR using the same primers as used in the Q-PCR. The reactions were performed using 20 µl (final volume) mixtures containing Faststart Taq DNA Polymerase PCR Buffer 1x, 2 µgram bovine serum albumin, 2.5mM MgCl_2_, 0.8mM of each deoxynucleoside triphosphate, 0.9 µM of each primer, and 1U of FastTaq DNA polymerase (Roche, Branford, CT, USA). The initial denaturation step was 95°C for 10 min, followed by 10 cycles of denaturation at 95°C for 15 s, annealing at 50°C for 60 s, and extension at 72°C for 60 s, followed by a final extension step that consisted of 72°C for 10 min.

The PCR products were purified using the Nucleospin Extract II kit (Machery-Nagel and quantified using the Quant-iT dsDNA Assay Kit on a Qubit fluorometer (Invitrogen).

Emulsion PCR was performed according to the protocol (emPCR Method Manual – Lib-A SV jan-2010) supplied with the GS FLX Titanium XLR 70 Sequencing kit (Roche, Branford, CT, USA).

**Data Analysis**

*Pyrosequencing and data-processing*

Amplicons were sequenced using the Roche / 454 Life Sciences Genome Sequencer FLX (Roche, Branford, CT, USA) pyrosequencing platform. Data analysis was performed using the software “Quantitative Insights into Microbial Ecology” (QIIME)[3]. Reads were removed if they were <200 and >500 nucleotides (nt) in length, if there were mismatches in the barcodes or primers, if ambiguous nucleotides were present or if the read quality score was <25. Chimeric sequences were filtered out using ChimeraSlayer [4]. Our data was denoised using the QIIME denoiser program [5]. Then we clustered the sequences into Operational Taxonomical Units (OTU’s) based on 97% sequence similarity (Uclust). The resulting OTU table was then condensed by removing all OTUs representing less than 0.005% of the total number of sequences [6]. Uclust classifier was used to assign taxonomy. [7]. The sequences were aligned using PyNast [8]. Sequences were rarefied (to remove the heterogeneity of the number of sequences per sample) prior to calculation of alpha and beta diversity statistics. Alpha diversity indexes were calculated in QIIME from rarefied samples using for diversity the Shannon index [9] for richness the Chao1 index [10]. Beta diversity was calculated using weighted and unweighted UniFrac [11] and principal coordinate analysis (PCoA) performed. Neighbour joining with nearest neighbour interchange phylogenetic trees were created using the representative sequences of each OTU and FastTree version 2.1.3 [12].

**REFERENCES**

1. Spear GT, Gilbert D, Sikaroodi M, Doyle L, Green L, Gillevet PM, Landay AL, Veazey RS: **Identification of rhesus macaque genital microbiota by 16S pyrosequencing shows similarities to human bacterial vaginosis: implications for use as an animal model for HIV vaginal infection.** *AIDS Res Hum Retroviruses* 2010, **26**:193–200.

2. Lane DJ, Pace B, Olsen GJ, Stahl DA, Sogin ML, Pace NR: **Rapid determination of 16S ribosomal RNA sequences for phylogenetic analyses.** *Proc Natl Acad Sci U S A* 1985, **82**:6955–9.

3. Caporaso JG, Kuczynski J, Stombaugh J, Bittinger K, Bushman FD, Costello EK, Fierer N, Peña AG, Goodrich JK, Gordon JI, Huttley GA, Kelley ST, Knights D, Koenig JE, Ley RE, Lozupone CA, McDonald D, Muegge BD, Pirrung M, Reeder J, Sevinsky JR, Turnbaugh PJ, Walters WA, Widmann J, Yatsunenko T, Zaneveld J, Knight R: **QIIME allows analysis of high-throughput community sequencing data.** *Nat Methods* 2010, **7**:335–6.

4. Haas BJ, Gevers D, Earl AM, Feldgarden M, Ward D V, Giannoukos G, Ciulla D, Tabbaa D, Highlander SK, Sodergren E, Methé B, DeSantis TZ, Petrosino JF, Knight R, Birren BW: **Chimeric 16S rRNA sequence formation and detection in Sanger and 454-pyrosequenced PCR amplicons.** *Genome Res* 2011, **21**:494–504.

5. Reeder J, Knight R: **Rapid denoising of pyrosequencing amplicon data: exploiting the rank-abundance distribution**. *Nat Methods* 2010, **7**:668–669.

6. Bokulich NA, Subramanian S, Faith JJ, Gevers D, Gordon JI, Knight R, Mills DA, Caporaso JG: **Quality-filtering vastly improves diversity estimates from Illumina amplicon sequencing.** *Nat Methods* 2013, **10**:57–9.

7. Edgar RC: **Search and clustering orders of magnitude faster than BLAST.** *Bioinformatics* 2010, **26**:2460–1.

8. Caporaso JG, Bittinger K, Bushman FD, DeSantis TZ, Andersen GL, Knight R: **PyNAST: a flexible tool for aligning sequences to a template alignment**. *Bioinformatics* 2010, **26**:266–267.

9. Shannon CE: **The mathematical theory of communication. 1963.** *MD Comput* 1963, **14**:306–17.

10. Chao A, Shen T: **Nonparametric estimation of Shannon’s index of diversity when there are unseen species in sample**. In *Environmental and Ecological Statistics*. *Volume 10*; 2003:429–443.

11. Lozupone C, Hamady M, Knight R: **UniFrac – An online tool for comparing microbial community diversity in a phylogenetic context**. *BMC Bioinformatics* 2006, **7**:371.

12. Price MN, Dehal PS, Arkin AP: **FastTree 2--approximately maximum-likelihood trees for large alignments**. *PLoSOne* 2010, **5**:e9490–.
